# Severe Hyponatremia From Syndrome of Inappropriate Antidiuretic Hormone Secretion as the Initial Presentation of a Gonadotroph Pituitary Neuroendocrine Tumor: A Case Report

**DOI:** 10.7759/cureus.105907

**Published:** 2026-03-26

**Authors:** Daniel Balderrama, Angélica N Murillo

**Affiliations:** 1 Internal Medicine, Hospital Metropolitano, Monterrey, MEX; 2 Internal Medicine, Programa Multicéntrico de Especialidades Médicas del Tecnológico de Estudios Superiores de Monterrey, Monterrey, MEX

**Keywords:** gonadotroph adenoma, hypogonadotropic hypogonadism, hyponatremia, pituitary adenoma, pituitary neuroendocrine tumor, sellar mass, syndrome of inappropriate antidiuretic hormone (siadh)

## Abstract

Pituitary neuroendocrine tumors, formerly referred to as pituitary adenomas, are common intracranial neoplasms. Most are benign and slow-growing. Clinical manifestations typically arise from hormonal hypersecretion or mass effect on adjacent structures. Hyponatremia due to syndrome of inappropriate antidiuretic hormone secretion is an uncommon presentation of pituitary tumors and has been rarely reported.

We present the case of a 35-year-old woman with a history of secondary amenorrhea and infertility who exhibited progressive headache and recurrent presyncope episodes. Laboratory tests showed that the patient had severe hyponatremia (sodium {Na}: 121 mmol/L), low serum osmolality (252 mOsm/kg), inappropriately elevated urine osmolality (748 mOsm/kg), elevated urinary sodium (112 mEq/L), hypouricemia (1.9 mg/dL), and a fractional excretion of uric acid of 11.66%, consistent with syndrome of inappropriate antidiuretic hormone secretion. Hormonal assessment indicated hypogonadotropic hypogonadism characterized by diminished levels of follicle-stimulating hormone, luteinizing hormone, and estradiol. Magnetic resonance imaging of the brain showed a sellar mass measuring 19 × 31 × 32 mm with a suprasellar extension and compression of the optic chiasm. Visual field testing corroborated bitemporal hemianopsia. The patient had an endoscopic transsphenoidal resection of the lesion. Histopathological examination identified a plurihormonal mature pituitary neuroendocrine tumor of gonadotroph lineage, exhibiting positive immunohistochemical staining for SF-1 and INSM1, negative expression for Pit-1, and a Ki-67 proliferation index of 10%.

This case illustrates an uncommon manifestation of a gonadotroph pituitary neuroendocrine tumor presenting as severe hyponatremia secondary to syndrome of inappropriate antidiuretic hormone secretion. Identifying this unusual presentation is crucial, as prompt diagnosis and intervention may avert considerable neurological, metabolic, and visual complications.

## Introduction

Pituitary neuroendocrine tumors, formerly known as pituitary adenomas, constitute about 10%-15% of all intracranial neoplasms and rank among the most prevalent primary brain tumors [1]. These lesions are generally benign and exhibit slow growth; however, they can lead to considerable morbidity based on their size, location, and hormonal activity.

Pituitary neuroendocrine tumors are categorized based on hormone production and cellular lineage, determined by transcription factor expression identified through immunohistochemistry, including Pit-1, T-PIT, and SF-1 [2,3]. Functioning tumors secrete excess hormones, resulting in particular endocrine syndromes, while non-functioning tumors typically exhibit symptoms associated with mass effect or hypopituitarism [4,5].

Gonadotroph tumors are the most prevalent subtype of non-functioning pituitary tumors and often remain asymptomatic until they attain significant dimensions, typically manifesting with visual disturbances, headache, or hypopituitarism [5,6].

Hyponatremia linked to pituitary tumors is predominantly associated with secondary adrenal insufficiency or hypothyroidism [7]. On the other hand, syndrome of inappropriate antidiuretic hormone secretion, which has been linked to pituitary tumors, is uncommon and has been documented in only a few instances [4,8]. We present a case of a gonadotroph pituitary neuroendocrine tumor that initially manifested with severe hyponatremia resulting from syndrome of inappropriate antidiuretic hormone secretion.

This case is particularly relevant due to the rare presentation of severe hyponatremia secondary to syndrome of inappropriate antidiuretic hormone secretion as the initial manifestation of a gonadotroph pituitary neuroendocrine tumor, an association that has been infrequently reported in the literature. Reporting such cases may improve early recognition and diagnostic approaches in similar clinical scenarios.

## Case presentation

A 35-year-old woman with no significant medical history presented in January 2026 with a progressive headache and recurrent presyncope episodes over the past month. She had a history of secondary amenorrhea and infertility and had been getting hormonal therapy for reproductive purposes.

On physical examination, the patient was hemodynamically stable with no focal neurological deficits aside from visual field impairment. No signs of volume depletion or edema were observed. There was no history of diuretic use or recent fluid imbalance. Differential diagnosis included non-functioning pituitary adenoma, craniopharyngioma, and other sellar masses.

The initial laboratory test showed severe hyponatremia, with a serum sodium (Na) level of 121 mmol/L. Further biochemical evaluation revealed low serum osmolality (252 mOsm/kg), elevated urine osmolality (748 mOsm/kg), and elevated urinary sodium excretion (112 mEq/L). Other results showed hypouricemia (1.9 mg/dL) and a fractional excretion of uric acid of 11.66%, consistent with syndrome of inappropriate antidiuretic hormone secretion (Table 1).

**Table 1:** Laboratory findings on admission

Test	Result	Reference Range
Serum sodium (mmol/L)	121	135-145
Serum osmolality (mOsm/kg)	252	275-295
Urine osmolality (mOsm/kg)	748	<100
Urinary sodium (mEq/L)	112	<40
Uric acid (mg/dL)	1.9	2.4-6
Fractional excretion uric acid (%)	11.66	<10

An endocrine test showed hypogonadotropic hypogonadism with follicle-stimulating hormone at 1.86 mU/mL, luteinizing hormone at 0.3 IU/mL, and estradiol at 22 pg/mL. Prolactin levels were slightly high at 27.1 ng/mL. Normal thyroid and adrenal function tests helped exclude hypothyroidism and adrenal insufficiency as potential causes of hyponatremia with thyroid-stimulating hormone at 1.8 mIU/L, free thyroxine at 1.2 ng/dL, and cortisol at 16.4 µg/dL (Table 2).

**Table 2:** Hormonal evaluation FSH, follicle-stimulating hormone; LH, luteinizing hormone; TSH, thyroid-stimulating hormone; T4, thyroxine

Test	Result	Reference Range
FSH (mU/mL)	1.86	3-10
LH (IU/mL)	0.3	2-15
Estradiol (pg/mL)	22	30-400
Prolactin (ng/mL)	27.1	4-23
TSH (mIU/L)	1.8	0.4-4.0
Free T4 (ng/dL)	1.2	0.8-1.8
Cortisol (µg/dL)	16.4	6-23

**Figure 1:**
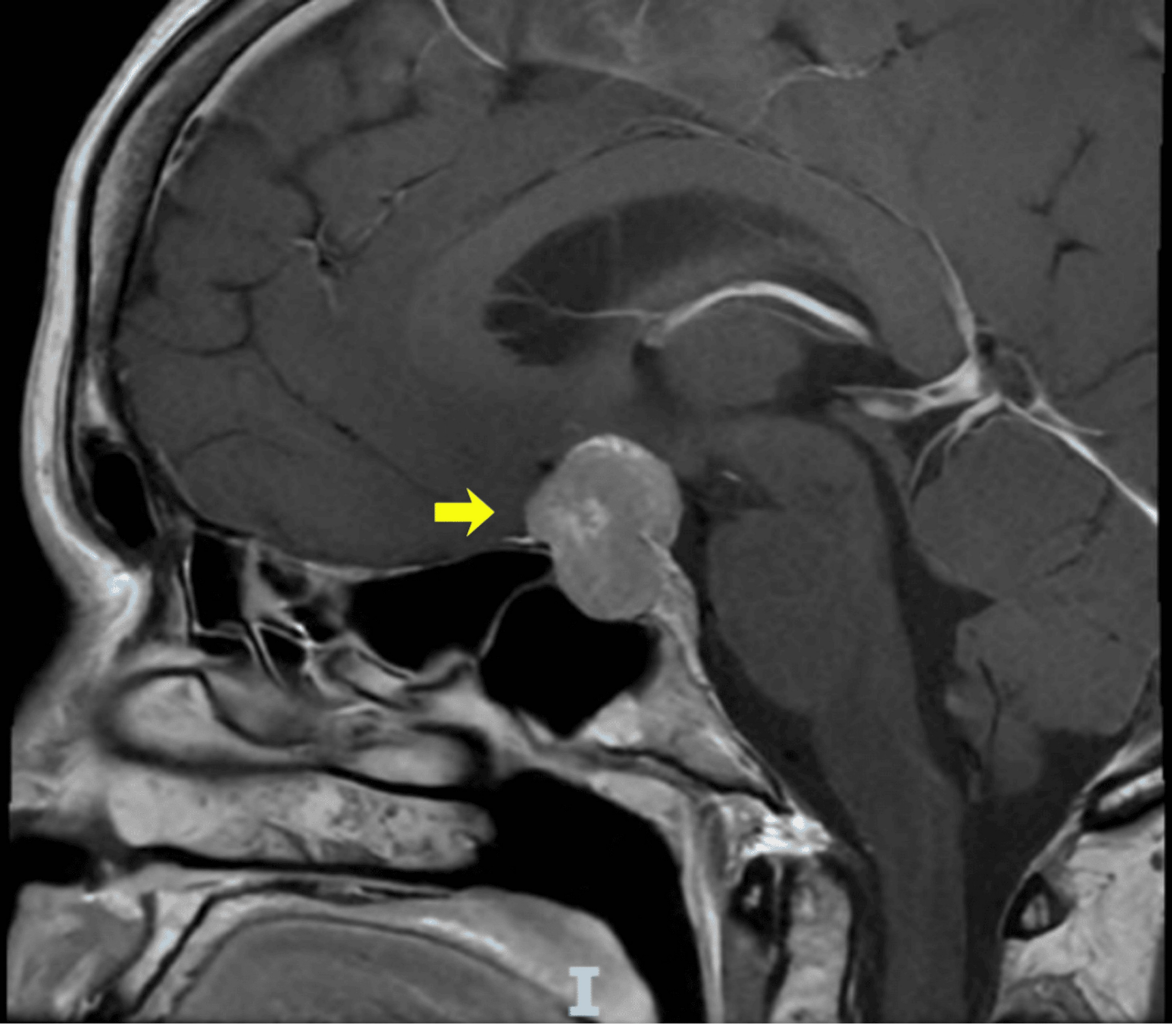
Sagittal T1-weighted contrast-enhanced magnetic resonance imaging Sagittal T1-weighted contrast-enhanced magnetic resonance imaging showing a sellar mass measuring 19 × 31 × 32 mm, appearing isointense on T1-weighted sequences with heterogeneous contrast enhancement and suprasellar extension, causing the compression of the optic chiasm (yellow arrow)

**Figure 2:**
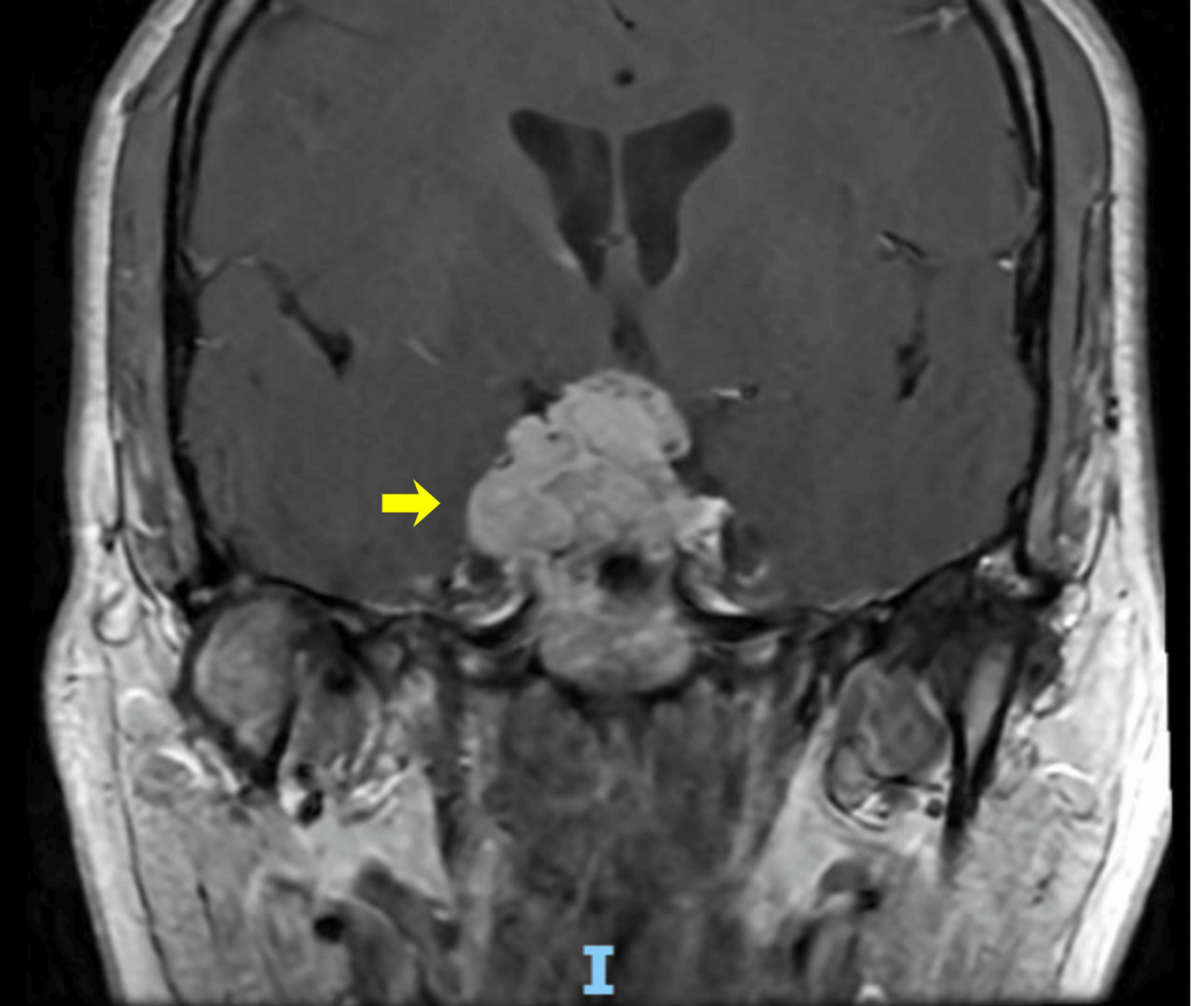
Coronal contrast-enhanced magnetic resonance imaging Coronal contrast-enhanced magnetic resonance imaging demonstrating a pituitary mass with heterogeneous enhancement and suprasellar extension, with the compression of the optic chiasm (yellow arrow)

Magnetic resonance imaging of the brain revealed a sellar mass measuring 19 × 31 × 32 mm with a suprasellar extension and compression of the optic chiasm. The lesion appeared isointense on T1-weighted images and demonstrated heterogeneous enhancement following contrast administration. It showed predominantly isointense to mildly hyperintense signal on T2-weighted sequences, with bilateral parasellar extension encasing the right internal carotid artery (Figures 1, 2).

Formal visual field testing demonstrated bitemporal hemianopsia, with a visual field index of 53% in the left eye and 62% in the right eye.

The initial therapy consisted of fluid restriction and oral salt supplementation to correct the hyponatremia. The patient subsequently underwent an endoscopic transsphenoidal surgical resection of the tumor without intraoperative complications. In the immediate postoperative period, serum sodium levels improved progressively with normalization following fluid management. No surgical complications were observed. No evidence of diabetes insipidus was observed postoperatively.

Histopathological analysis identified a plurihormonal mature pituitary neuroendocrine tumor of gonadotroph lineage. Immunohistochemistry showed that SF-1 and INSM1 were stained positively, Pit-1 was not stained, and the Ki-67 proliferation index was 10%.

During follow-up, the patient demonstrated clinical improvement with the resolution of presyncope and the stabilization of electrolyte levels.

## Discussion

Gonadotroph pituitary neuroendocrine tumors are the predominant subtype of non-functioning pituitary tumors and typically originate from gonadotroph cells that express the transcription factor SF-1 [2,9]. These tumors typically do not exhibit clinically significant hormone hypersecretion and are predominantly diagnosed due to mass effect, which may manifest as headaches, visual disturbances, or hypopituitarism.

Hyponatremia is infrequently linked to pituitary tumors. When present, it is most often attributed to secondary adrenal insufficiency resulting from adrenocorticotropic hormone deficiency or, less frequently, hypothyroidism [8]. Conversely, syndrome of inappropriate antidiuretic hormone secretion linked to pituitary tumors is uncommon and has been documented in only a few cases in the literature [8,10].

The pathophysiological mechanism connecting pituitary tumors to syndrome of inappropriate antidiuretic hormone secretion is not fully elucidated. Proposed mechanisms include the impairment of hypothalamic control over antidiuretic hormone secretion due to suprasellar extension, the compression of the pituitary stalk, or hypothalamic irritation caused by tumor growth [6,7,10].

In this patient, the presence of suprasellar extension and the compression of adjacent structures may have resulted in the dysregulation of antidiuretic hormone secretion.

The present World Health Organization classification designates pituitary adenomas as pituitary neuroendocrine tumors, indicating their neuroendocrine differentiation and diverse biological behavior [2]. The presence of SF-1 and INSM1 in immunohistochemistry and the absence of Pit-1 expression confirm the lineage of gonadotrophs. The Ki-67 proliferation index of 10% is also higher than what is usually seen [2]. Higher Ki-67 levels have been linked to more aggressive tumor behavior and may require more careful monitoring after surgery [2,11].

This case further expands the clinical spectrum of gonadotroph pituitary neuroendocrine tumors, demonstrating that severe hyponatremia due to syndrome of inappropriate antidiuretic hormone secretion may precede the diagnosis of a sellar mass. The recognition of this unusual presentation is important, as early neuroimaging in patients with unexplained syndrome of inappropriate antidiuretic hormone secretion and neurological symptoms may facilitate early diagnosis and treatment [11,12].

Although syndrome of inappropriate antidiuretic hormone secretion has been described in association with intracranial tumors, its occurrence in pituitary neuroendocrine tumors remains rare [8,12]. Previous reports suggest that suprasellar extension and hypothalamic involvement may play a role in antidiuretic hormone dysregulation [7,12]. Similar to previously reported cases, our patient presented with severe hyponatremia and radiological evidence of suprasellar extension, supporting this proposed mechanism. However, the presence of hypogonadotropic hypogonadism as an associated finding further highlights the complexity of pituitary dysfunction in these tumors.

This case underscores the importance of considering intracranial pathology in patients presenting with unexplained hyponatremia, particularly when accompanied by neurological symptoms.

## Conclusions

Gonadotroph pituitary neuroendocrine tumors are typically non-functioning lesions and manifest with symptoms associated with mass effect or hypopituitarism. However, syndrome of inappropriate antidiuretic hormone secretion may represent a rare initial manifestation.

This case highlights a rare presentation of a gonadotroph pituitary neuroendocrine tumor manifesting as severe hyponatremia due to syndrome of inappropriate antidiuretic hormone secretion. The combination of biochemical abnormalities and suprasellar mass effect underscores the importance of recognizing atypical presentations of pituitary tumors. The early identification of this uncommon association may facilitate timely diagnosis and management, potentially preventing neurological and visual complications.
